# Comparative study of macrophages in naked mole rats and ICR mice

**DOI:** 10.18632/oncotarget.19661

**Published:** 2017-07-28

**Authors:** Jishuai Cheng, Zheng Yuan, Wenjing Yang, Chang Xu, Wei Cong, Lifang Lin, Shanmin Zhao, Wei Sun, Xiaosong Bai, Shufang Cui

**Affiliations:** ^1^ Laboratory Animal Centre, Second Military Medical University, Shanghai, China; ^2^ Department of Science and Technology, Academy of Military Medical Sciences, Beijing, China; ^3^ School of Kinesiology, Shanghai University of Sport, Shanghai, China; ^4^ Department of Clinical Laboratory, Shi Dong Hospital, Shanghai, China

**Keywords:** naked mole rats, ICR mice, spleen, macrophage, immune response

## Abstract

The domestic and foreign scholars have studied naked mole rats more focused on the respect such as its long life, resistant to low oxygen, little spontaneous tumor, but the study of the immune system is little. In this study, we compared the anatomy and tissue morphology of NMR and ICR mouse spleens and found that the gross appearance of the NNMR spleen differed from ICR. There were more macrophages in NNMR spleens than in ICR spleens. Furthermore, we focused on the differences of macrophages. We compared their phagocytic capabilities and the data showed that NNMR macrophages are more phagocytic than ICR mouse macrophages. We also used polyI:C and LPS to stimulate the NMR and ICR macrophages and then measured the immune response as expression of certain TLR signaling molecules. After stimulation, there was a lower increase in apoptosis of NMR macrophages than ICR macrophages and a non-significant increased expression of TLRs in NMR macrophages than in ICR macrophages. In contrast, NF-κB proteins increased more significantly in NMR’s than in ICR’s and the expression of downstream cytokines in NMR macrophages also increased more than in ICR macrophages. Based on these results, we hypothesize that in addition to being able to eat foreign matter, NMR macrophages can activate the TLRs, start the NF-κB and produce a large number of cytokines to enhance immune response, so as to protect the body from outside interference when the virus or bacteria invading.

## INTRODUCTION

*Heterocephalus glaber*, the naked mole rat (NMR), is a prevalent underground rodent in Africa. In 1967, Jarvis first brought the NMRs into the laboratory for scientific research. Since then, more biologists have begun to cooperate with Jarvis for naked mole rat biological research [1–7]. While the research objectives with naked mole rats have been increasing, most have confirmed the existing theories of anti-cancer, anti-aging, and low oxygen resistance properties in NMRs. Studies of the NMR immune system are scarce, and research about their immune cells is lacking [8–11].

Immune system is an important index of biomedical research, which not only has a guiding significance for animal physiological state judgments and feeding management, also is the important basis of clinical medical treatment and scientific research. Immune system which is the most effective weapon defense pathogens invasion consists of immune organs, immune cells and immune molecules, and it can find and remove foreign bodies, exogenous pathogenic microorganisms, etc. There have been studies to uncover the link between the immune system and cancer. Along with the aging of the body, the immune system is gradually deteriorating, which is immune senescence. And immune senescence is related to systemic inflammation, chronic inflammatory diseases and many cancers.

The spleen is the body’s largest lymphoid organ, which has important functions including immunity, hematopoiesis, and filtering blood [12]. The spleen is the main place to produce the antibodies in the primary immune response and hematogenous antigens in immune response, while the spleen can produce promoting consume peptide, complement, tumor necrosis factor and so on which are related factors of humoral immunity.

Macrophages are important immune cells in the body that mediate and maintain innate or acquired immune defense mechanisms [13], at the same time are also important antigen presenting cells (APCs) that aid in mediating immune responses. When participating in the innate immune response, macrophages phagocytize and kill invading microbes and produce signaling molecules to strengthen the acute inflammatory response. They promote antigen presentation and regulate T cell responses in the acquired immune response [14]. In addition to phagocytosis, chemotaxis, adhesion, and antigen presentation, macrophages also play a role in the secretion of cytokines.

Herein, we started a pilot study to compare the immune system of NMRs to mice’s. First, we compared the spleens of NMRs and mice at the histological and cellular levels. We found that the proportion of NMR splenic macrophages was greater than that observed in mouse spleens. We also demonstrated that naked mole rat macrophages had greater phagocytic capability than mouse macrophages. We also profiled the immune response of NMR and mouse macrophages after stimulation with mimics of viral and bacterial pathogens. We found that NMR macrophages protected themselves from pessimal stimulation by secreting high levels of cytokines. In accordance with this data, we speculate that macrophages of NMRs play an important role in the innate immune response.

## RESULTS

### Comparative studies: differences in the anatomy and tissue morphology of NMR and ICR mouse spleens

**Figure 1:**
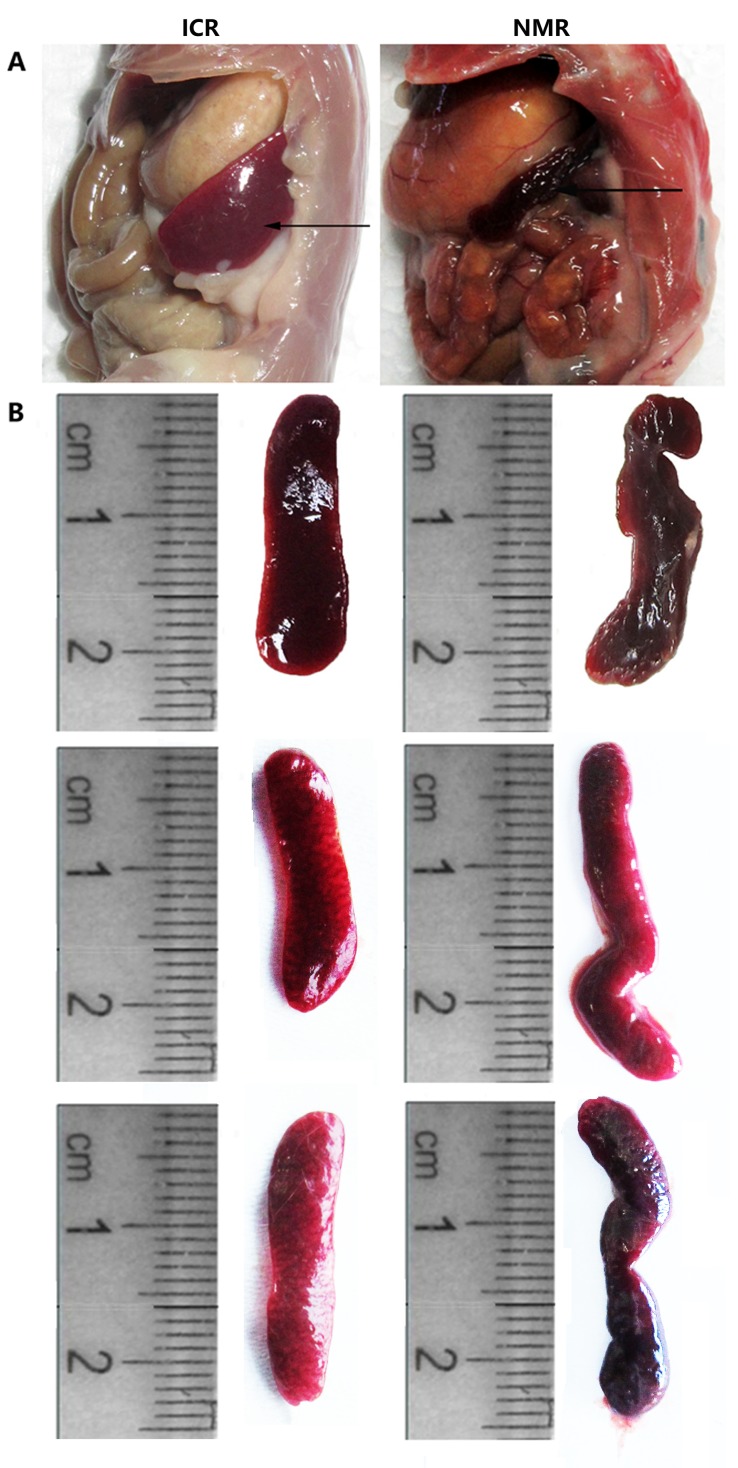
Differences in tissue anatomy characteristics between NMR and ICR mouse spleens **(A)** The representative images show the spleen anatomical location in ICR mice and NMRs. **(B)** The pictures show the spleen appearance in ICR mice and NMRs.

We first compared the anatomical structure of the spleens of naked mole rats and mice. Through anatomical observation, we found that the mouse spleen was located in the left rib arch, the majority of it hidden behind the stomach, and its shape was smooth, flat, and oval (Figure 1A). The mouse spleen had purple coloration, flexible texture, and an average length of approximately 2.3 cm (Figure 1B). The naked mole rat spleen was located in the abdominal cavity behind and below the stomach, bordering the cecum. NMR spleens had a slender and irregular shape, light purple color, soft texture, were more flexible than mouse spleen, and had an average length more than 2.5 cm (Figure 1A-1B). These results suggest that the anatomical and morphological structures of the spleen in naked mole rats and mice have notable differences.

**Figure 2:**
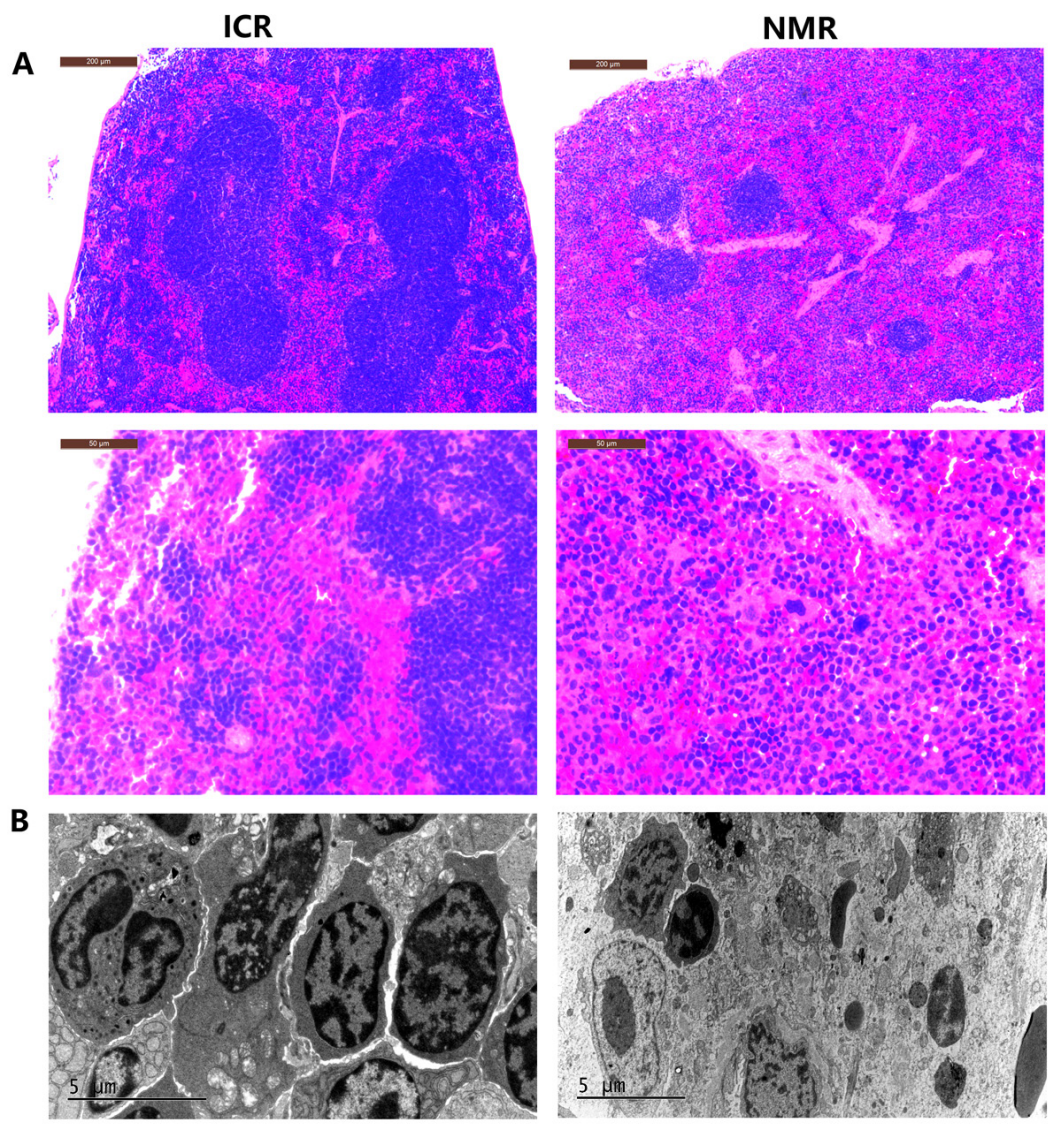
Differences in tissue morphology characteristics between NMR and ICR mouse spleens **(A)** The representative images show H&E staining of spleens in ICR mice and NMRs (10s, 40n magnification). **(B)** The pictures show the splenic structure in ICR mice and NMRs using scanning electron microscopy techniques.

We also compared the shape and structure of NMR and mouse spleens. By microscopy, the red pulp distribution area in the mouse spleen was small. The mouse splenic corpuscle area was large and had a concentrated distribution. Mice had fewer splenic trabeculae, which were short and thin (Figure 2). In naked mole rat spleens, the red pulp distribution area was large. The splenic corpuscle was small and located between the red pulps with a dispersed concentration. The spleen trabeculae were greater in number, thicker, and longer than those of mice. The NMR red pulp and marginal zone were rich in macrophages (Figure 2). The results showed that, compared with mice, naked mole rat red pulp and spleen trabeculae were more developed, and the NMR red pulp and marginal zone contained more red blood cells and macrophages. Macrophages are immune cells that have important immune functions. A higher abundance of macrophages may allow an organism to mount a more effective immune response against exogenous factors to better protect the body.

### NMR macrophages have higher phagocytosis than ICR mouse macrophages

**Figure 3:**
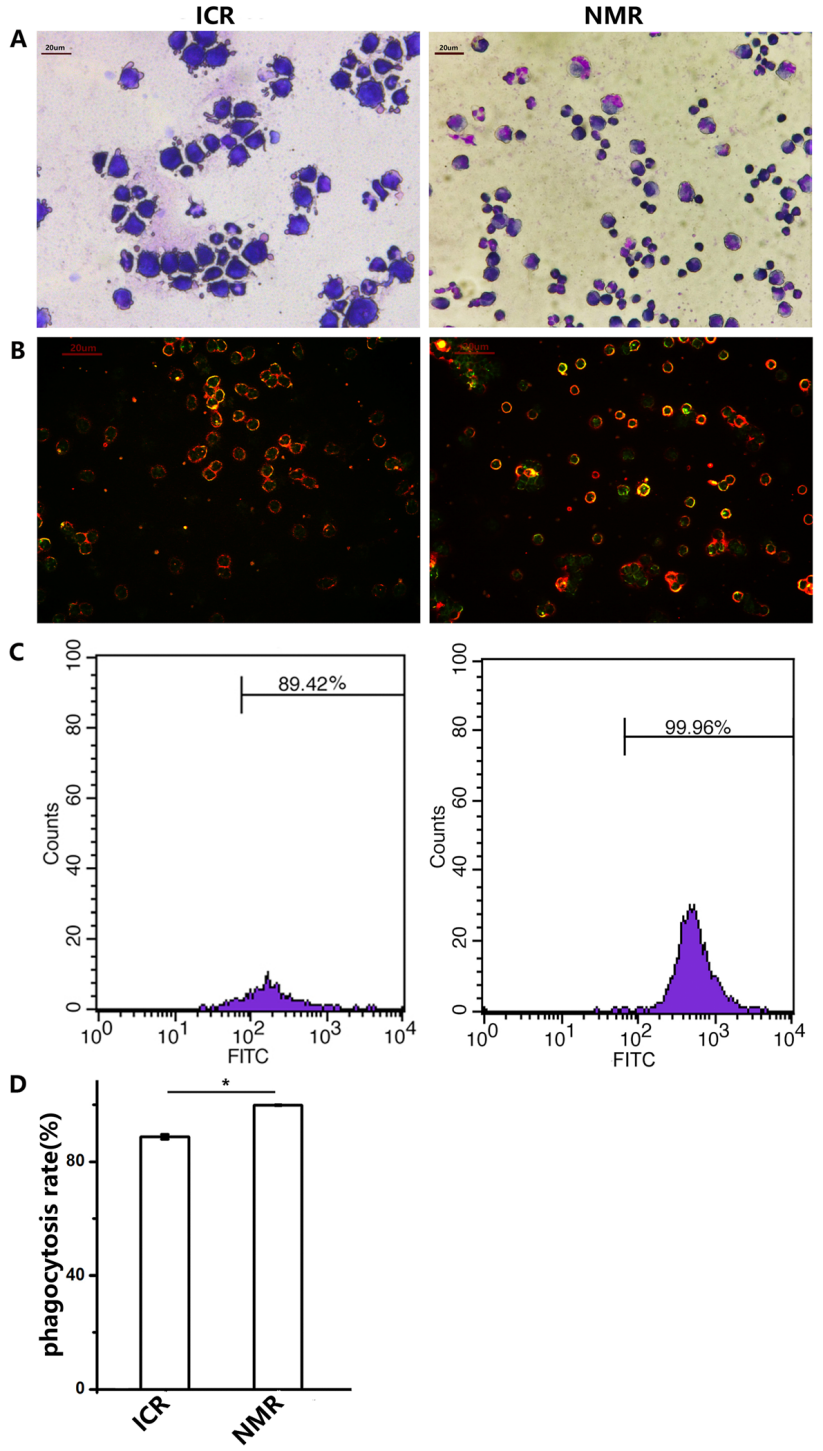
Naked mole rat macrophages have higher phagocytic capabilities than those of ICR mice **(A)** The representative images show the macrophages of ICR mice and NMRs engulfing chicken red blood cells (63× magnification). **(B)** Macrophages in ICR mice and NMRs take up FITC-Dextran. **(C,D)** The FCM (flow cytometry) results of phagocytosis in ICR and NMR macrophages. The results are presented as the means±SD (^*^P<0.05, independent-samples t test).

As macrophages usually phagocytose foreign bodies, we examined the phagocytic functions of NMR and mouse macrophages. At first, we microscopically observed ICR and NMR macrophages engulfing chicken red blood cells. Over the same time course, NMR macrophages swallowed more chicken red blood cells than ICR macrophages (Figure 3A). We simultaneously used fluorescein-labeled glucan to verify the phagocytic function. Then, we examined the phagocytosis of naked mole rat and ICR macrophages by flow cytometry (Figure 3B-3C). We found that the phagocytosis rate of naked mole rat macrophages was up to 99.81±0.13%, but the phagocytosis rate of ICR mice macrophages was only 88.79±0.90%. These results suggested that the phagocytosis rate of naked mole rat macrophages was higher than that of ICR mouse macrophages.

### Naked mole rat macrophages have lower apoptosis than those of ICR mice after polyI:C and LPS exposure

**Figure 4:**
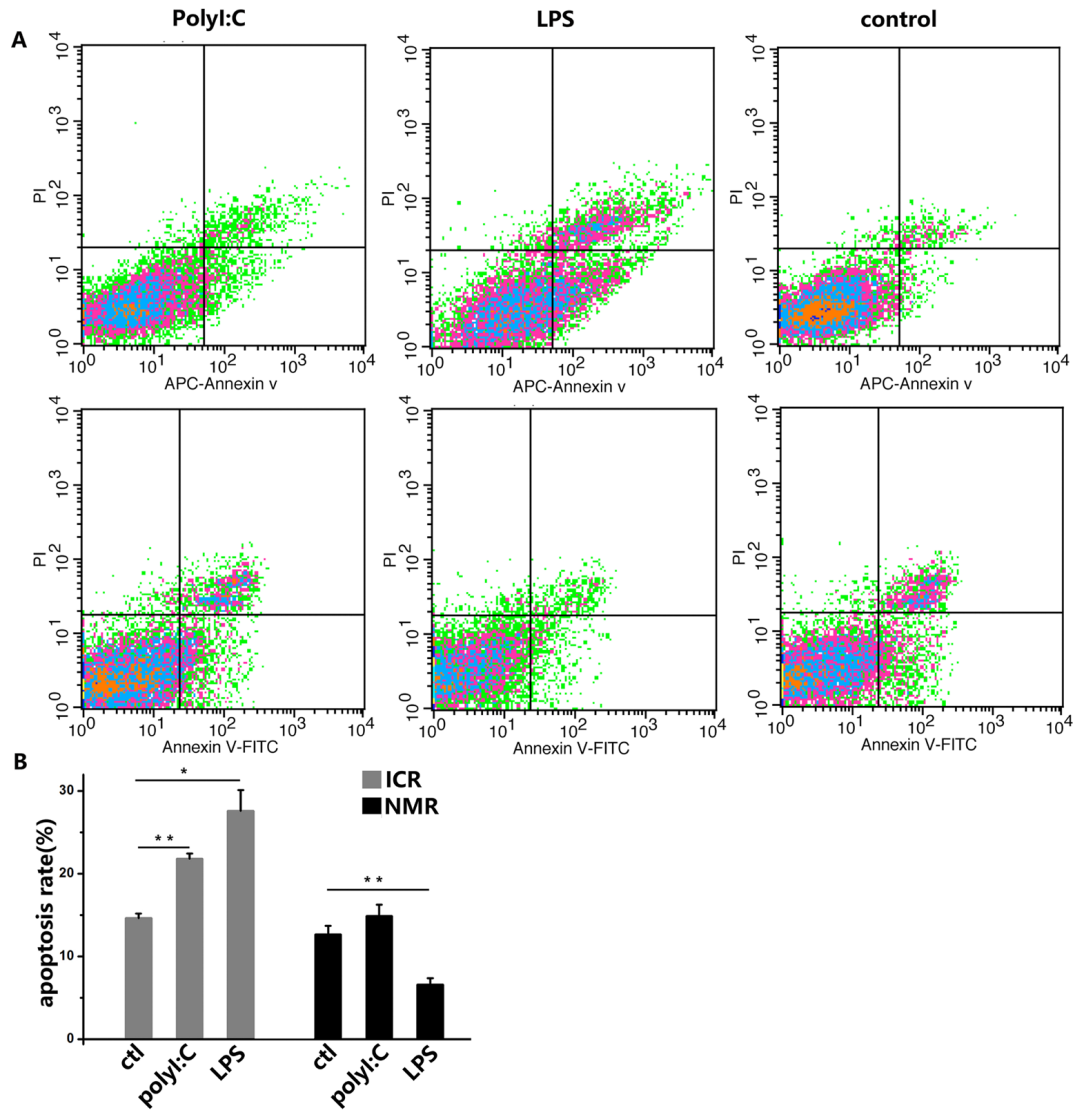
PolyI:C and LPS induce apoptosis in mouse and naked mole rat macrophages **(A)** Macrophages were treated with 10 ng/ml polyI:C or 1 ng/ml LPS for 12 h. The negative control was treated with PBS. Apoptosis was analyzed with PI and annexin V staining by flow cytometry. The upper panels represent the results for mice, and the lower panels correspond to naked mole rats. **(B)** The graph indicates the percentage of apoptotic cells (means ± SD). ^*^P<0.05, ^**^P<0.01.

In addition to our study of phagocytosis by macrophages in naked mole rats and ICR mice, we tested the influence of apoptosis when macrophages were stimulated with analogs of viral and bacterial infections. Polyinosinic acid-polycytidylic acid (polyI:C) is a synthetic double-stranded RNA that is often used to simulate an infection because its structure is similar to the RNA of a variety of viruses [15]. Lipopolysaccharide (LPS), the main component of Gram-negative bacterial cell walls, has very strong immunogenicity and can enhance immune responses [16]. Therefore, macrophages were treated with polyI:C or LPS. In the flow cytometry assay of ICR mouse macrophages, 6.82% more cells in the polyI:C-treated group and 16.19% more cells in the LPS-treated group were PI-positive or annexin-V-positive, indicative of apoptotic cells. However, in naked mole rat macrophages, there was only 2.24% more cell apoptosis in the polyI:C-treated group. For the LPS-treated NMR macrophages, the apoptosis rate significantly declined (Figure 4A, 4B).

### Naked mole rat macrophages are protected from polyI:C challenge

**Figure 5:**
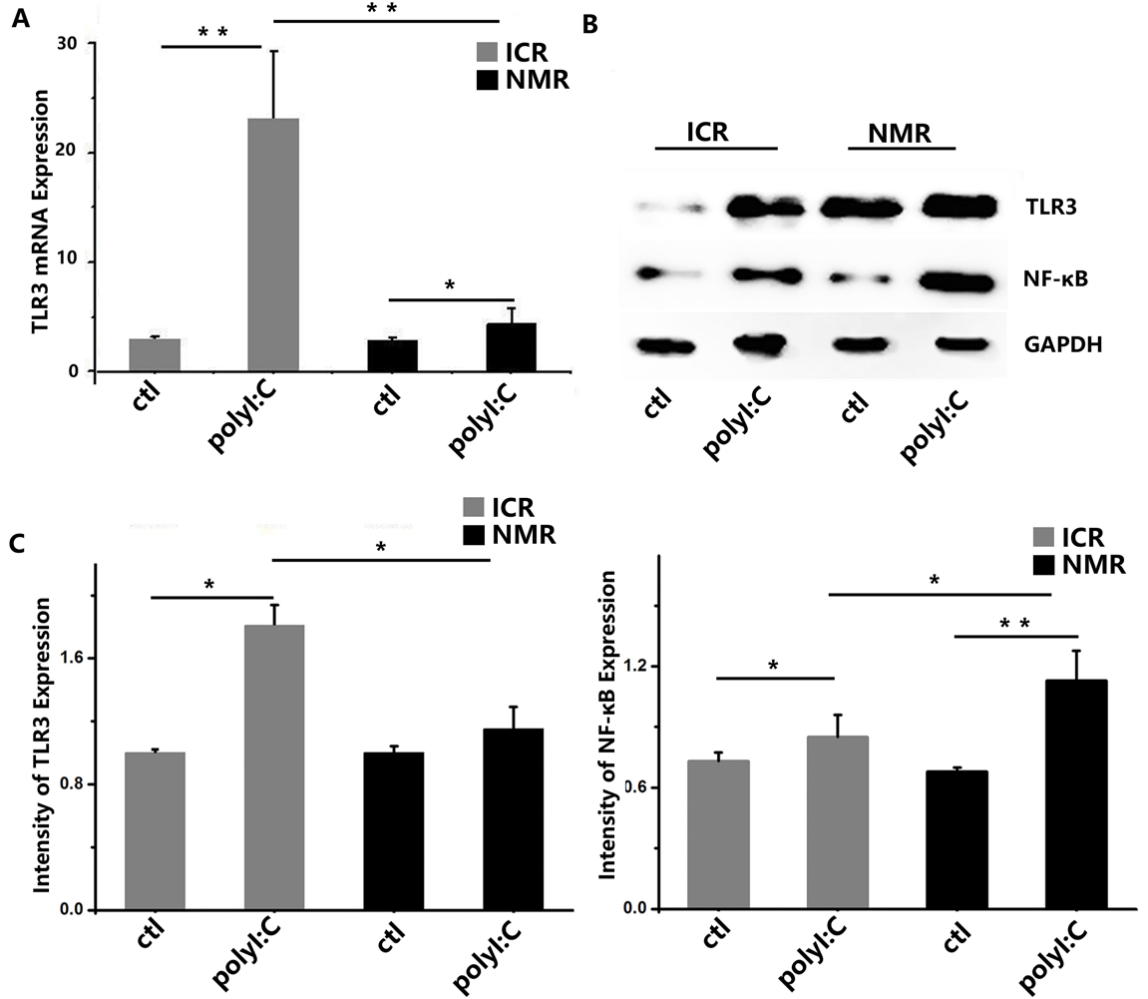
PolyI:C-stimulated macrophage inflammatory responses in naked mole rats and ICR mice **(A)** Toll-like receptor 3 mRNA expression levels in macrophages were measured in both naked mole rats and ICR mice. After treatment, mouse and NMR macrophages showed increased TLR3 mRNA expression. **(B)** Western blot analysis of TLR3 and NF-κB in macrophages taken from ICR mice and NMRs before or after treatment. **(C)** Western blot analysis of TLR3 and NF-κB in macrophages taken from ICR mice and NMRs before or after treatment. Results of (A, C) are presented as the means±SD (^*^P<0.05, ^**^P<0.01, independent-samples t test).

We used polyI:C to simulate virus infection and studied the macrophage inflammatory or immune responses. Using real-time PCR, toll-like receptor 3 (TLR3) expressions significantly increased in ICR mouse macrophages and naked mole rat macrophages after treatment. There was a significant difference in TLR3 expression between ICR mouse macrophages and NMR macrophages. The expression levels of IFN-β in both ICR and NMR macrophages were significantly elevated, and the level was increased more in NMRs than in ICR mice (Figure 5A). In mouse macrophages, the relative TLR3 mRNA expression was 20.84±6.57, but it was only 2.39±1.49 in NMRs. We also used Western blotting for protein expression to further verify the real-time PCR results. We found that TLR3 protein expression in ICR mouse macrophages was strongly increased by polyI:C. In NMRs, there was no clear difference in response to polyl:C, and there were significant differences between ICR mouse macrophages and NMR macrophages (Figure 5B). We also tested the protein expression of nuclear factor-kappa B (NF-κB), a key member of the NF-κB signaling pathway. According to the Western blot data, NF-κB protein expression was significantly increased in ICR mouse macrophages compared to controls, while the rise in NMR macrophages was modest. Similar to the case of TLR3, there were significant differences between ICR mice and NMRs (Figure 5C).

### Naked mole rat macrophages are protected from LPS challenge

**Figure 6:**
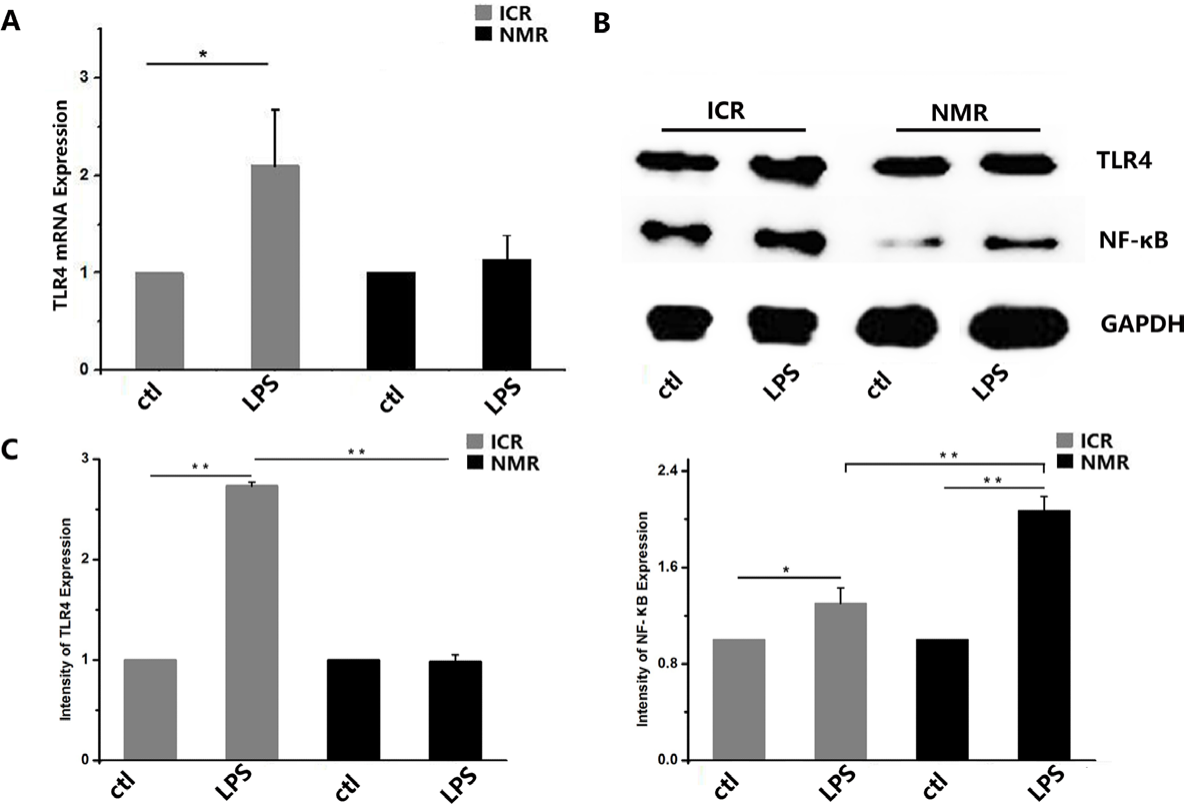
LPS-stimulated inflammatory responses in naked mole rat and ICR mouse macrophages **(A)** Toll-like receptor 4 mRNA expression levels were measured in both naked mole rats and ICR mice. After treatment, ICR mouse and NMR macrophage TLR4 mRNA expression increased. **(B)** Western blot analysis of TLR4 and NF-κB in macrophages taken from ICR mice and NMRs before or after treatment. **(C)** Western blot analysis of TLR4 and NF-κB in macrophages taken from ICR mice before or after treatment. Results of (A,C) are presented as the means±ea (^*^P<0.05,^**^P<0.01, independent-samples t test).

We used LPS to simulate bacterial infection and studied the inflammatory immune responses of macrophages. Using real-time PCR, we found that TLR4 expression in ICR mouse macrophages increased after treatment, and the naked mole rat macrophages also showed significantly increased expression. There was no difference in TLR4 expression between ICR mouse macrophages and NMR macrophages. The expression levels of TNF-α in both ICR mouse and NMR macrophages were extremely significantly elevated, and the levels increased more in NMRs than in ICR mice (Figure 6A). In mouse macrophages, the relative TLR4 mRNA expression was 2.10±0.97, and it was 2.41±0.68 in NMRs. We also used Western blotting for protein expression analysis to further verify the real-time PCR results. We found that TLR4 protein expression in ICR mouse macrophages was extremely increased. However, the TLR4 protein levels in NMRs were almost unchanged, and there were significant differences between ICR and NMR results (Figure 6B). We also tested the protein expression of NF-κB. According to the Western blotting results, NF-κB protein expression in ICR mouse macrophages significantly increased, and it was extremely increased in NMR macrophages. Similarly, there were significant differences between ICR mice and NMRs (Figure 6C).

### Comparative studies: differences in secretion of cytokines between NMR and ICR mouse macrophages

**Figure 7:**
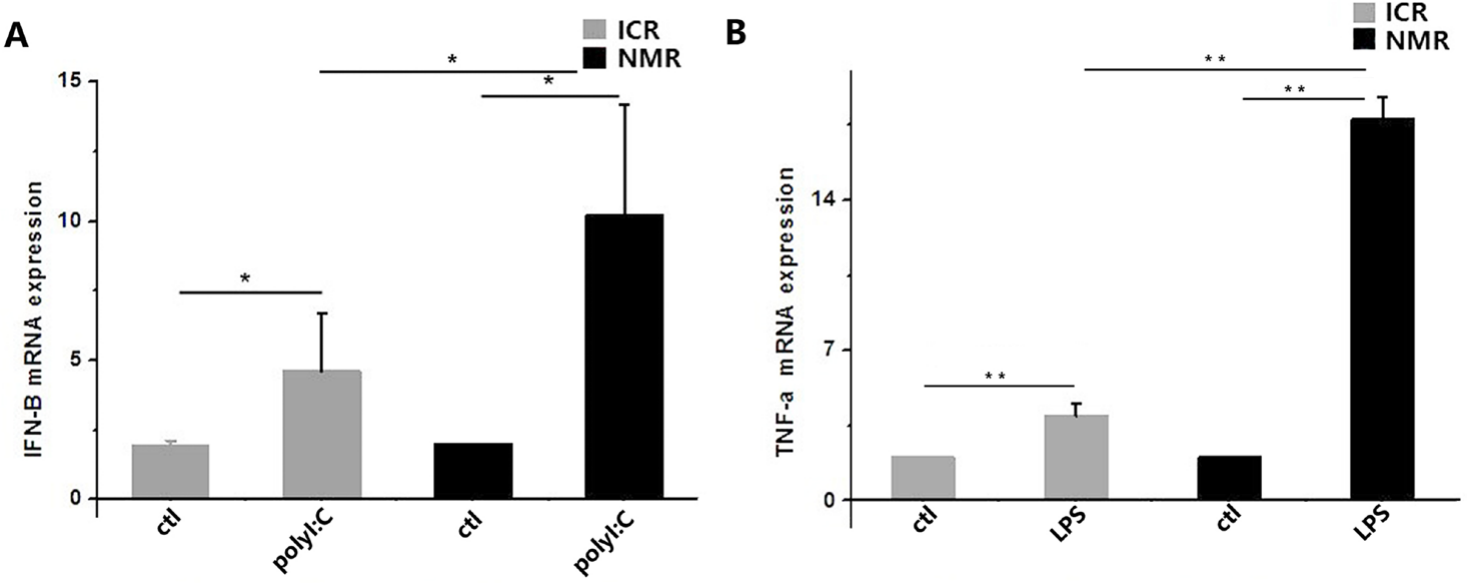
PolyI:C- and LPS-stimulated macrophage cytokine expression in naked mole rats and ICR mice **(A)** Interferon-β mRNA expression levels in macrophages were measured in both naked mole rats and ICR mice. Both types of macrophages had increased IFN-β mRNA expression. **(B)** Tumor necrosis factor-α mRNA expression levels in macrophages were measured in both naked mole rats and ICR mice. Macrophage TNF-α expression was significantly increased. The results of (A,B) are presented as the means±ea (^*^P<0.05,^**^P<0.01, independent-samples t test).

Our results indicated that Toll-like receptors in macrophages were activated when the cells were stimulated with polyI:C or LPS. PolyI:C acts through TLR3, and LPS mainly plays a role through TLR4 to activate NF-κBα, thus promoting the transcription and expression of cytokines. We measured cytokines by real-time PCR: we detected IFN-β in the polyI:C-treated group and TNF-α in the LPS-treated group. The expression of IFN-β increased in ICR mouse macrophages by 3.61±2.08, but it increased to 9.20±4.01 in NMR macrophages (Figure 7A). The expression of TNF-α increased in ICR mice macrophage by 2.95±0.56, while it significantly increased to 16.74±1.07 in NMR macrophages (Figure 7B). There was a significant difference in ICR mouse macrophages and naked mole rat macrophages.

## DISCUSSION

Naked mole rats have the longest life span of all rodents. As a group, the oldest individuals are more than 30 years old, and they are in generally good health in their 20s. Those who are 25 or older show some signs of weakness and most NMRs eventually die of old age. Some studies have suggested animal models of longevity are able to tolerate a broad spectrum of harmful stimuli. Compared with mice, naked mole rat cells have stronger resistance to many harmful insults (e.g., paraquat, high temperature, heavy metals, DNA damaging agents, and harmful foreign material) [8–11]. These studies indicate that NMRs have a stronger ability to resist disease damage at the cellular level over the course of their lives, but the underlying mechanisms are unclear. Our team imported naked mole rats from Africa in 2011, bred them in an artificial environment, and conducted NMR biological researchs [17–21].

The spleen, the largest lymphoid organ of the body, has many important functions in immunity, hematopoiesis, and blood filtration. The spleen is the main site of IgM antibody production, especially in the primary immune response. The spleen can also promote phagocytosis of peptides, complement, tumor necrosis factor, and many kinds of humoral factors. The white pulp is composed of dense lymphoid cells, and it is critical for maintaining the specificity of the immune response. Blood flow in the red pulp is slow, and it is the main site of phagocytosis by immune cells. There are more macrophages in the marginal zone, and these cells are important for identifying and capturing antigens and inducing immune responses [22, 23]. In this study, we compared the anatomy and tissue morphology characteristics of NMR and ICR mouse spleens. We found major differences in their appearances. H&E staining revealed that naked mole rat spleen capsules and trabeculae are thicker. The inside of the smooth muscle and elastic fibers are developed, and the spleen and trabecular smooth muscle and elastic fibers in the telescopic adjustable spleen and differences in volume suggest that the spleens of NMRs may be enhanced by the ability to adjust blood volume. The NMR splenic corpuscle structure is more complete and can produce more plasma cells, which is conducive to the synthesis and secretion of IgG. In NMR spleens, the density of B cells was lower than in the mouse spleen, but the number of macrophages was increased. The main function of B cells in the spleen is to produce immunoglobulin which is immune-mediated by body fluids. We speculate that NMR spleen humoral immunity mediated by B cells is weak and that NMR spleen mainly takes part in nonspecific immune response through phagocytosis and takes participate in specific immune response by T cell-mediated immune response.

Macrophages are an important immune cell of the body, and they play a central role in the immune response. Macrophages play an important role in regulating innate and acquired immune defense mechanisms, and they are the main scavenger cells for responses to viral and bacterial infections, acting by engulfing and digesting pathogenic microorganisms to remove pathogenic materials. Macrophages are also antigen presenting cells that mediate immune responses [24, 25]. In the article, westudied macrophages by the detection of their phagocytic function and the response to polyI:C and LPS. After polyI:C and LPS stimulation, the TLRs in NMR macrophages had a non-significant increase in comparison to in ICR macrophages, but the expression of NF-κB and cytokines had remarkably elevated. Toll-like receptors (TLRs) are a type of pattern recognition receptor (PRR) that can identify bacterial or viral pathogen-associated molecular patterns (PAMPs). They can signal through the bone marrow response protein (MyD88) signaling pathway, activating other intracellular signaling pathways. TLRs can activate NF-κB, which is a transcription factor that regulates many genes and participates in cellular proliferation, apoptosis, invasion, differentiation, pro-inflammatory factor secretion (e.g., interferon, tumor necrosis factor, etc.), and inflammation [26]. TLR3 participate in the identification of double-stranded RNA virus. After TLR3 is activated, the body’s cells synthesise and release a series of cytokines and inflammatory mediators by activating the NF-κB, causing the activation of antiviral protein, such as interferon to play its antiviral effect. After TLR4 feeling the PAMP stimulation of invading pathogens, it ultimately entered into the nucleus by intracellular signaling pathways, activating NF-κB in the nuclear genes and corresponding mRNA transcription, synthesis of interleukin, TNF, IFN and other cytokines and release them into the extracellular, resulting in early immune response.

In the article, we studied the differences between ICR mouse macrophages and NMR macrophages by the detection of their phagocytic function and the response to polyI:C and LPS. We found that naked mole rat macrophages had increased phagocytic capability compared to mouse macrophages. After polyI:C and LPS stimulation, the TLRs in NMR macrophages had a non-significant increase in comparison to in ICR macrophages, but the expression of NF-κB and cytokines had remarkably elevated. These results suggest that a small amount of TLR activation in NMR macrophages can cause high expression of downstream NF-κB and increased expression of cytokines, resulting in a strong immune response to protect the body from damage by viruses and bacteria. Based on the results of our experiments, we hypothesize that the high phagocytic capability of naked mole rat macrophages is a powerful tool to resist invasion of exogenous substances. This phagocytic capability may be a factor that contributes to the long lifespan and low rate of spontaneous tumors in NMRs. There are many other cells, such as B cells, T cells, and dendritic cells, in immune system. What these cells function in the immune response of NMR and how these immune cells interact remain to be seen.

**Table:** Sheet 1 Nucleotide Sequence Specific Primers

Gene	Primer sequences		Species
TLR3	5’CCTTGTTGGGACTGTGGC3’	5’GGCAGGTGGCAATCTTCT3’	NMR
TLR4	5’GAGCCGCTGGTGTATCTT3’	5’CAGGGACTTCTCCAACTTTT3’	NMR
IFN-β	5’ACAACGCAGCAGCAGTTT3’	5’GAGCAGCATTCTCCTTCC3’	NMR
TNF-α	5’ATGGCATGGATCTAACGG3’	5’CGGCTGACAGTATGGGTG3’	NMR
GAPDH	5’CGCCTGCTTCACCACCTT3’	5’CCTGCCGCCTGGAGAAA3’	NMR
TLR3	5’AGACTGATGCTCAGGAGGGTG3’	5’GAAGAGGGCGGAAAGGTG3’	ICR
TLR4	5’TTGATACTGACAGGAAACCCTA3’	5’TTGTCTCCACAGCCACCA3’	ICR
IFN-β	5’ATCCCTATGGAGATGACG3’	5’ATGGCAAAGGCAGTGTAA3’	ICR
TNF-α	5’CACCACGCTCTTCTGTCT3’	5’ATTTGGGAACTTCTCATCC3’	ICR
GAPDH	5’CATGGCCTTCCGTGTTCCTA3’	5’GCGGCACGTCAGATCCA3’	ICR

## MATERIALS AND METHODS

### Animals and reagents

ICR mice (6-8-week-old) were obtained from Joint Ventures Sipper BK Experimental Animals (Shanghai, China). Mice were kept and bred in pathogen-free conditions. Naked mole rats (over 10 months) were bred by the Second Military Medical University Laboratory Animal Center (Shanghai, China). All animal experiments were undertaken in accordance with the National Institutes of Health Guide for the Care and Use of Laboratory Animals with approval of the Scientific Investigation Board of Second Military Medical University (Shanghai, China). PolyI:C and LPS were from Sigma-Aldrich (Darmstadt, Germany). Antibodies specific to TLR3, TLR4, NF-κB and GAPDH used for immunoblotting were from Proteintech (Chicago, IL, USA).

### Sample preparation and observation

After intraperitoneal injection of 0.3% sodium pentobarbital anesthetic, the abdominal cavity was opened for spleen removal and measurement. Spleens were placed in 10% neutral formalin; fixed for 24 hours; and subsequently dehydrated, paraffin embedded, sectioned, H&E stained, and imaged using optical microscopy to record morphological structure characteristics.

### Cell culture

Naked mole rats were sacrificed using cervical dislocation and soaked for 2-3 min in 15% ethanol. Under sterile conditions, the femur and tibia were separated, cartilage tissue was removed, the bone marrow cavity was rinsed using a 1 ml syringe with complete medium, and cells were processed into a single cell suspension. After centrifugation, red blood cells were lysed using red blood lysis fluid, and complete medium was added to end lysis. After adding complete medium, the heavy suspension cells were cultured for 8 to 12 hours at 37°C in 3% O_2_, 5% CO_2_, and 92% N_2_. After collecting and centrifuging the supernatant, cells were suspended using DMEM containing 60 ng/ml M-CSF and allowed to develop for 6 ∼ 7 d. The status of the cells was observed by an inverted microscope. Mouse cells were maintained at 35°C in Dulbecco’s Modified Eagle’s Medium with high glucose (DMEM; GIBCO, USA) supplemented with 10% fetal bovine serum (FBS; GIBCO, USA) and 100 units/mL penicillin/streptomycin in a humidified incubator with 5% CO_2_ and 21% O_2_. NMR cells were maintained at 35°C in Dulbecco’s Modified Eagle’s Medium with low glucose (DMEM; GIBCO, USA) supplemented with 10% fetal bovine serum (FBS; GIBCO, USA) and 100 units/mL penicillin/streptomycin in a humidified incubator under conditions of 92% N_2_, 5% CO_2_, and 3% O_2_.

### Phagocytosis test

Macrophages were kept at room temperature away from light and incubated with fluorescent particles (FITC-Dextran) for 30 min. After PBS washing, we used flow cytometry to test the phagocytosis by each type of macrophage [27].

### Cell apoptosis assay

An Annexin V-FITC assay [28] was used to quantify numbers of apoptotic cells by flow cytometry according to the manufacturer’s instructions (Nanjing Keygen Biotech, KGA108). Briefly, cells were collected following trypsinization, washed twice with ice-cold PBS, and resuspended in 300 μL of 1× binding buffer containing 5 μL Annexin V-FITC and 5 μL PI for 30 min at room temperature in the dark. All samples were analyzed on a FACSCalibur flow cytometer (BD Biosciences).

### RNA isolation and real-time PCR to assess gene expression

GAPDH, TLR3, TLR4, IFN-β and TNF-α mRNA levels in the macrophages from naked mole rats and ICR mice were quantified by real-time PCR. Total RNA was extracted with RNAiso Plus (Takara, Dalian, China) [29] according to the manufacturer’s instructions. RNA quantity and purity were assessed by A260/A280 absorbance. First-strand cDNA was generated from total RNA with the TIANScript cDNA First-Strand Kit (Tiangen, Beijing, China). The transcript expression levels were quantified with the StepOnePlus Real-time PCR Detection System (Applied Biosystems, Warrington, UK) using the SYBR® Green RT-PCR kit (Takara, Dalian, China). Other mRNA transcript levels were then normalized to the expression of GAPDH transcripts. To allow the comparison of mRNA expression, the real-time PCR data were analyzed with the ΔΔCt method and normalized to the amount of GAPDH cDNA, the endogenous control.

### Protein extraction and western blot analysis

At the end of treatment, fibroblasts were lysed in lysis buffer (50 mM Tris-HCl, pH 7.4, 150 mM NaCl, 1 mM EDTA, 1 mM EGTA, 1μg/mL protease inhibitor cocktail, 5 mM phenylmethylsulfonyl fluoride, and 1 mM dithiothreitol containing 1% Triton X–100). Lysates were centrifuged at 10,000× g for 10 min at 4°C, and protein concentration was determined. Samples (50μg/lane) were resolved by 10% SDS-polyacrylamide gel electrophoresis and transferred onto a polyvinylidene fluoride (PVDF) membrane. Blots were blocked for 1 h at 37°C in 20 mM Tris-HCl, pH 7.4, 150 mM NaCl, and 0.02% Tween-20 (TBST) containing 5% skimmed milk and probed using a 1:1000 dilution of the appropriate primary antibodies (anti-TLR3, TLR4, NF-κB, and GAPDH) for overnight incubation at 4°C. The blots were washed thrice with TBST and probed with goat-anti-mouse IgG or goat-anti-rabbit IgG conjugated to horseradish peroxidase (1:2000, Wuhan Boster Biotech, Wuhan, China). Quantitative analyses of protein band optical intensities were conducted using the Kodak Gel Logic 4000R Imaging System (Carestream, USA) and normalized to GAPDH for protein expression or to total protein for protein phosphorylation [15].

### Statistical analysis

Results are given as the means plus or minus standard deviation (SD). Comparisons between two groups were performed using Student’s t test. Statistical significance was determined as P values less than 0.01.
